# Carbon Dots in Photodynamic/Photothermal Antimicrobial Therapy

**DOI:** 10.3390/nano14151250

**Published:** 2024-07-25

**Authors:** Siqi Wang, Colin P. McCoy, Peifeng Li, Yining Li, Yinghan Zhao, Gavin P. Andrews, Matthew P. Wylie, Yi Ge

**Affiliations:** School of Pharmacy, Queen’s University Belfast, 97 Lisburn Road, Belfast BT9 7BL, UK; swang54@qub.ac.uk (S.W.);

**Keywords:** carbon dots, nanoparticles, photosensitizers, photodynamic therapy, photothermal therapy, antimicrobial

## Abstract

Antimicrobial resistance (AMR) presents an escalating global challenge as conventional antibiotic treatments become less effective. In response, photodynamic therapy (PDT) and photothermal therapy (PTT) have emerged as promising alternatives. While rooted in ancient practices, these methods have evolved with modern innovations, particularly through the integration of lasers, refining their efficacy. PDT harnesses photosensitizers to generate reactive oxygen species (ROS), which are detrimental to microbial cells, whereas PTT relies on heat to induce cellular damage. The key to their effectiveness lies in the utilization of photosensitizers, especially when integrated into nano- or micron-scale supports, which amplify ROS production and enhance antimicrobial activity. Over the last decade, carbon dots (CDs) have emerged as a highly promising nanomaterial, attracting increasing attention owing to their distinctive properties and versatile applications, including PDT and PTT. They can not only function as photosensitizers, but also synergistically combine with other photosensitizers to enhance overall efficacy. This review explores the recent advancements in CDs, underscoring their significance and potential in reshaping advanced antimicrobial therapeutics.

## 1. Introduction

In recent years, the global challenge of antimicrobial resistance (AMR) has escalated significantly [1,2]. As pathogens continue to develop resistance against standard treatments, there is an urgent need for innovative therapeutic approaches. Traditional antibiotics are gradually losing their effectiveness, prompting researchers to investigate alternative solutions.

Among the alternatives, photodynamic therapy (PDT) and photothermal therapy (PTT) have emerged as notable contenders utilizing light. Historically, psoralens were employed with light for vitiligo treatments in ancient Egypt and India [3]. In 1903, Niels Finsen received a Nobel Prize for pioneering modern phototherapy [4]. The true potential of PDT, however, was unlocked in the 1960s through laser technology. The coupling of photosensitizers with lasers heralded a noninvasive approach to treating tumors [5] and bacterial infections [6]. Upon light exposure, these PSs initiate the production of reactive oxygen species (ROS), conferring a profound therapeutic effect by selectively targeting tumors and bacterial infections while mitigating adverse effects [7]. While PDT produces ROS to combat microbial cells, PTT uses heat to induce cellular damage. These approaches differ in their mechanisms for ROS generation, with PDT relying on two primary mechanisms. Conversely, in PTT, heat is harnessed—instead of oxygen—to target tumors or infections, also facilitating photoacoustic imaging. The efficacy of these approaches is predominantly contingent upon the photosensitizers, which absorb light to initiate the healing process.

Nanotechnology, the manipulation of matter at the nanoscale, has revolutionized various fields, including medicine and materials science. Recently, it has emerged as a powerful tool in the development of photosensitizers [8,9,10,11]. This burgeoning field harnesses the distinct properties of nanomaterials to enhance the efficacy and specificity of photosensitizers, offering numerous advantages over conventional approaches. By exploiting the tunable characteristics and high surface area to volume ratio of nanostructures, researchers can tailor photosensitizers to target specific cells or tissues with unprecedented precision [12,13,14,15,16]. More interestingly, nano-sized photosensitizers or photosensitizing nano-composites could be developed with enhanced photophysical properties [17], improved biocompatibility [18], and targeted delivery capabilities [19]. 

Carbon dots (CDs) have emerged as a highly versatile and promising class of nanomaterials with some distinctive properties that make them particularly noteworthy in the fields of PDT and PTT [20,21,22,23]. These nanoscale carbon-based particles, typically ranging from a few to tens of nanometers in size, have unique optical and chemical properties that make them highly attractive for biomedical applications. CDs possess tunable fluorescence [24,25,26], excellent photostability [27,28], low cytotoxicity, and high biocompatibility [29,30,31], rendering them ideal candidates for innovative therapeutic applications.

In the realm of PDT, CDs could serve as potent PSs, capable of generating reactive oxygen species (ROS) upon exposure to specific wavelengths of light. These ROS play a crucial role in inducing cytotoxic effects on targeted cells. Simultaneously, their ability to convert light into heat makes them valuable contributors to PTT, inducing cellular damage through elevated temperatures. This dual functionality of CDs in both PDT and PTT positions them as promising tools for the precise and efficient treatment of various diseases, ranging from cancer [32,33] to microbial infections [32,34]. Furthermore, due to the facile surface functionalization of CDs, they can be conjugated with other PSs to realize a synergistic effect in PDT and PTT [35,36,37]. As a result, the overall therapeutic efficacy can be enhanced while potentially minimizing side effects associated with traditional treatments.

In this review, we explore the recent advancements and capabilities of CDs in PDT and PTT, highlighting their potential to revolutionize the landscape of antimicrobial therapeutics and to inspire further research endeavors aimed at harnessing their full potential in combating infectious diseases.

## 2. Principles of PDT/PTT for Antimicrobial Therapy

The Jablonski Diagram serves as a visual representation of the fundamental processes underlying PDT [7]. PSs in their ground state (S_0_) absorb visible light photons, transitioning to their first electronically excited singlet state (S_1_). This state can further undergo intersystem crossing (ISC), flipping its spin and transitioning to the first excited triplet state (T_1_). Notably, the T_1_ state exhibits relatively long-lived characteristics, persisting in the millisecond range, facilitating subsequent energy and electron transfers. Deactivation from the S_1_ state may lead to fluorescence emission, which is valuable for diagnostic purposes. Alternatively, deactivation of the T_1_ state can result in phosphorescence emission or undergo thermal decay, dissipating the excited energy of the PS.

**Figure 1:**
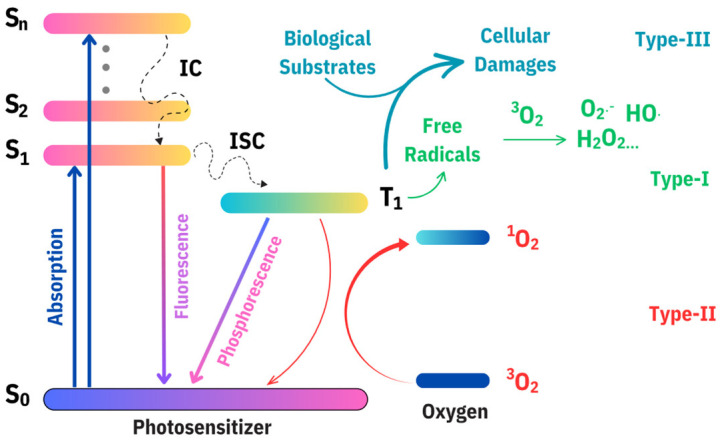
Schematic illustration of three types of mechanisms in photodynamic therapy (PDT) including a series of potential energy transfer processes that occur in photosensitizers after light excitation (Jablonski diagram), along with possible photodynamic therapy mechanisms (with (Type I and Type II) or without oxygen (Type III)) that may take place during this process. The Jablonski diagram illustrates the electronic states of a molecule, including the ground state (S_0_), singlet excited states (S_1_, S_2_, S_n_), and triplet states (T_1_). Upon absorption of a photon, electrons are excited from S_0_ to higher singlet states (S_1_, S_2_, Sn). Fluorescence occurs when electrons return from an excited singlet state (S_1_) to the ground state (S_0_). Intersystem crossing (ISC) is a non-radiative transition from a singlet state (S_1_) to a triplet state (T_1_). Phosphorescence involves the emission of a photon when electrons return from a triplet state (T_1_) to the ground state (S_0_). Internal conversion (IC) represents non-radiative transitions between singlet states (e.g., S_n_ to S_1_) [40,41].

As shown in Figure 1, three primary photoreactions, namely Type I, Type II, and Type III, govern the photodynamic process [38,39,40]. In Type I, the transfer of energy or electrons from activated PSs leads to the formation of ROS, including hydroxyl radicals (HO), superoxide anions (O_2_^−^), and hydrogen peroxide (H_2_O_2_). The Type II mechanism involves an interaction between the excited state of PSs and oxygen molecules, resulting in the production of reactive singlet oxygen (^1^O_2_) through direct energy transfer. This process highlights the crucial role of singlet oxygen generation on the efficacy of photodynamic reactions. In contrast, Type III PDT is characterized by activated PSs directly interacting with specific target biomolecules, such as DNA and proteins. Despite its potential, the stringent requirements of Type III PDT limit the suitability of many PSs for this application. Therefore, the discovery and research of photosensitizers based on the Type III mechanism are currently very limited. Among all these types, Type II PDT is the most extensively studied, with the production of ^1^O_2_ being a key indicator of a PS’s effectiveness; the more ^1^O_2_ produced, the more potent the photodynamic action. To measure ^1^O_2_, various techniques such as electron spin resonance (ESR), fluorescence, ultraviolet (UV)-visible spectroscopy, and time-resolved approaches like flash photolysis are employed. Notably, fluorescence and UV-visible spectroscopy play pivotal roles in quantifying the yield of ^1^O_2_.

For microbes, ROS can have several detrimental effects as follows: (1) damage to cell membranes: ROS can increase the permeability of the microbial cell membranes, ultimately leading to their lysis [42]; (2) disruption of enzymatic processes: ROS can oxidize essential enzymes, thereby impeding crucial microbial metabolic processes [43]; (3) nucleic acid damage: ROS-induced oxidative stress can result in breaks within DNA and RNA strands, hindering both replication and transcription processes [44].

As a comparison, in PTT, light energy undergoes rapid conversion into heat through processes that do not involve photon emission [45,46]. When light is absorbed, the energy propels the molecule into an excited state, from which it can follow a route known as internal conversion (IC). During internal conversion, the molecule transitions between different electronic energy levels without emitting photons. Despite descending to a lower energy level through IC, the molecule remains in an excited state relative to its original position. Subsequently, the molecule encounters two primary relaxation pathways. It may return to its ground state by emitting a photon, a process termed radiative decay, observable in phenomena such as fluorescence or phosphorescence. Alternatively, relaxation may occur without photon emission, referred to as non-radiative decay, which is common among photothermal agents [20]. In non-radiative decay, the energy initially absorbed from light is dissipated as vibrational energy. This vibrational energy is distributed across various molecular motions or modes, representing the diverse ways in which atoms within the molecule can oscillate or shift relative to each other. As the molecule interacts with neighboring molecules, it transfers this vibrational energy, resulting in an increase in kinetic energy and thereby raising the local temperature. It is vital to note that the efficiency of this light-to-heat conversion varies among molecules and materials. Factors such as molecular structure, environmental conditions, and the interplay between non-radiative and radiative processes play significant roles in determining this efficiency. In the context of PTT, selecting agents with a propensity for non-radiative decay is paramount [47,48]. This ensures that absorbed light energy is primarily converted into heat, facilitating effective heat generation.

For microbes, exposure to heat can result in several significant effects as follows: (1) protein denaturation: a key consequence of heat generated through PTT is the denaturation of proteins within microbial cells [49,50,51]. Elevated temperatures can disrupt the native conformation of proteins, rendering them inactive. This includes crucial cellular components such as structural proteins, enzymes, and membrane proteins. The loss of functional proteins can disrupt essential cellular processes, impairing microbial viability; (2) membrane disruption: cellular membranes, particularly lipid bilayers, are highly sensitive to temperature fluctuations. Increased heat from PTT can induce membrane fluidization and lipid peroxidation, leading to the eventual rupture of the cell membrane [52,53]. This disruption compromises membrane integrity, resulting in the leakage of intracellular contents. Ultimately, this process culminates in cell lysis, further reducing microbial survival; (3) vascular effects: in tumors or microbial colonies, the elevated temperatures from PTT can adversely affect nearby blood vessels [52,54]. This thermal damage can lead to constriction or even rupture of blood vessels, thereby impeding blood flow. Consequently, affected microbes experience nutrient deprivation and reduced oxygen supply, contributing to cellular stress and eventual cell death; (4) in addition to causing direct thermal damage, PTT can also induce cell death via apoptosis [55]. The elevated temperature from PTT initiates a cascade of intracellular pathways, resulting in the release of cytochrome c from the mitochondria and the activation of caspases, pivotal components in the apoptotic cell death process. Recent research indicates that PTT might also enhance the body’s immune response [56,57]. The demise of cells, such as tumor cells, under PTT conditions can prompt the release of tumor-associated antigens. Consequently, this release may stimulate the body’s immune system to identify and eliminate residual or metastatic cancer cells.

## 3. Photosensitizers

The concept of harnessing light to produce therapeutic effects dates back centuries. Ancient Egyptians and Indians were known to use light-sensitive plants combined with sun exposure to treat vitiligo [3]. However, the contemporary era saw a significant leap forward with the emergence of photosensitizers in the early 20th century, predominantly for cancer treatment [58]. The term “photodynamic therapy” was coined to describe this interaction between light and photosensitizer in the presence of oxygen, which often leads to cytotoxic outcomes. The earliest photosensitizers were derived from natural sources, such as hematoporphyrin, a precursor of heme [59]. Over time, scientific advancements enhanced our comprehension of photosensitizing mechanisms and facilitated their optimization for diverse therapeutic applications. Consequently, second- and third-generation photosensitizers emerged, distinguished by enhanced selectivity, reduced side effects, and improved tissue penetration [60].

### 3.1. Methylene Blue

Methylene blue (MB) has experienced a resurgence of interest as a potential photosensitizer, particularly in the latter half of the 20th century coinciding with the emergence of PDT as a therapeutic approach [61]. Functioning as a photosensitizer, MB can be activated by specific light wavelengths to generate ROS. These ROS, in turn, exhibit cytotoxic effects, notably targeting microbial pathogens and cancer cells. Within the realm of antimicrobial PDT, MB activated by light has demonstrated effectiveness against bacteria [62,63] and tumors [64,65]. MB offers a compelling array of advantages as a photosensitizer, including a well-established safety profile, affordability, and straightforward synthesis. However, certain limitations such as non-selectivity, restricted tissue uptake, rapid clearance, and the potential for resistance exist. Recently, efforts have focused on incorporating MB into nano- and microparticle platforms, promising to enhance the efficacy of PDT [66].

### 3.2. Porphyrin-Based Photosensitizers

Porphyrins have long been known to exhibit photo-reactivity. The observation of photosensitive skin in patients with porphyria, a group of disorders linked to porphyrin metabolism abnormalities, provided early insights into the photodynamic properties of these compounds. Hematoporphyrin, one of the first natural porphyrins used clinically, and its derivatives were among the earliest photosensitizers studied for PDT [67]. They were initially explored for tumor treatment but soon found applications in microbial inactivation [68]. Photofrin, developed as a more purified derivative of hematoporphyrin, made a significant breakthrough in the 1990s as the first PDT drug to receive market approval [69]. Another milestone came with the introduction of 5-Aminolevulinic acid in the same era, widely adopted due to its prodrug nature, converting into the potent photosensitizer protoporphyrin IX within cells [70]. Apart from those natural porphyrins and their derivatives, synthetic counterparts like tetraphenylporphyrins (TPPs) and their derivatives have been explored [71]. Notably, a novel XF porphyrin derivative has shown promise in combating methicillin-resistant Staphylococcus aureus [72] and Escherichia coli strains [73]. However, the potential toxicity concerns associated with TPPs necessitate thorough investigations before clinical implementation, possibly requiring tailored delivery systems or formulations to optimize biodistribution.

### 3.3. Phthalocyanines

Phthalocyanines (Pcs) possess a large planar structure closely related to the natural porphyrins [74]. The core of the molecule is composed of four isoindole units joined by nitrogen atoms, forming a cyclic conjugated system that is highly delocalized. Their structure can be modified with various substituents, allowing for fine-tuning of their photophysical properties, solubility, and cellular uptake. In addition to the metal-free phthalocyanines just with a basic phthalocyanine structure, there are different phthalocyanines photosensitizers functionalized with different metal atoms or functional groups, such as metallophthalocyanines (e.g. zinc phthalocyanine (ZnPc) [75] and aluminum phthalocyanine [76]), silicon phthalocyanine [77], as well as substituted phthalocyanines (e.g., sulfonated phthalocyanines [78] and lipophilic phthalocyanines [79]). The introduction of axial ligands to metallophthalocyanines allows for modulation of their aggregation behavior, photophysical properties, and pharmacokinetic profiles [80]. Additionally, Pcs can be conjugated with other molecules or platforms to enhance targeting, improve delivery, or introduce supplementary functionalities, such as peptide-conjugated Pcs [81] and polymer-conjugated Pcs [82].

### 3.4. Natural Dyes

Natural dyes have a rich historical legacy, dating back centuries when they were primarily used to color textiles and for various other applications [83]. Sourced from plant, animal, or mineral sources, these dyes have now found new applications in therapeutic realms, leveraging their capacity to generate ROS upon exposure to light [84]. Among the notable natural dyes today are curcumin, hypericin, and riboflavin (vitamin B2). 

For example, curcumin, which is extracted from the turmeric plant, has garnered significant attention due to its well-documented antimicrobial [85], anti-inflammatory [86], and antioxidant [87] properties. Notably, curcumin’s potential as a photosensitizer has recently gained prominence, with applications extending to its use as a carbon source in the development of PDT and PTT functional CDs [88,89]. In a recent study reported by Pal et al. [90], a novel surface-passivated carbon dot derived from curcumin and polyethyleneimine was synthesized using a straightforward one-step hydrothermal method. Compared to pure curcumin, the resulting CDs exhibited significantly improved aqueous solubility, offering a promising approach to enhancing curcumin’s solubility. Moreover, these CDs demonstrated effective bacterial labeling and antioxidant activity.

Derived from St. John’s Wort (hypericum perforatum), hypericin has also demonstrated efficacy against a variety of microbes, including antibiotic-resistant strains, when activated by light [91]. Riboflavin is a water-soluble vitamin, essential for energy production and the metabolism of fats, drugs, and steroids. Acting as a photosensitizer, riboflavin absorbs light and transfers energy to molecular oxygen, thereby generating ROS [92].

### 3.5. Fullerenes

Fullerenes, commonly referred to as “buckyballs”, are a class of carbon allotropes composed entirely of carbon, taking the form of a hollow sphere, ellipsoid, or tube. C60, a spherical molecule with 60 carbon atoms, is the most well-known fullerene, although various derivatives and related structures, such as C70, exist. It is worth noting that fullerenes exhibit a remarkable capacity to absorb a wide spectrum of light, particularly within the UV and visible ranges. Upon absorption, they undergo intersystem crossing, leading to the formation of triplet excited states. These states can subsequently interact with molecular oxygen, giving rise to singlet oxygen and other ROS, rendering fullerenes highly effective photosensitizers [93]. 

### 3.6. Up-Conversion Nanoparticles and Nanoplatforms

Both the natural and synthetic photosensitizers, as discussed previously, not only exhibit some favorable photosensitive properties but also are capable of mitigating the risk of drug resistance. However, the prevalent drawbacks of most of those photosensitizers persist, namely poor water solubility and limited depth of penetration. As a solution, the advent of up-conversion nanoparticles (UCNPs) has addressed these issues, marking a significant advancement in the field. UCNPs leverage near-infrared (NIR) light, which boasts superior tissue penetration compared to visible light, thereby broadening the scope of PDT [94]. 

Various nanomaterials, including lanthanide-doped nanoparticles [95], gold nanoparticles (AuNPs) [96], graphene oxide [97], and quantum dots [98], have emerged as efficient candidates for PDT/PTT. Lanthanide ions, owing to their 4f-4f electronic transitions, offer sharp emission bands and long luminescence lifetimes [99]. AuNPs, on the other hand, excel in light absorption and scattering through localized surface plasmon resonance (LSPR), generating robust electromagnetic fields near their surfaces [100]. Moreover, the resonance wavelength of AuNPs can be finely tuned by manipulating their size, shape, and environment, rendering them highly adaptable for therapeutic purposes [100,101]. When UCNPs are in proximity to AuNPs, the local electromagnetic field enhancement, because of the LSPR of AuNPs, can amplify the luminescence intensity of UCNPs [102]. This enhancement can improve the efficiency of photosensitizer activation. Meanwhile, the energy upconverted from UCNPs can be transferred to gold nanoparticles, enabling the generation of heat through the photothermal effect or enhancing the production of reactive oxygen species, thus amplifying the PDT effect [103].

Quantum dots (QDs) are semiconductor nanocrystals that possess unique photophysical properties. By adjusting their size, the emission wavelength of QDs can be easily tailored, offering customization according to specific therapeutic requirements [104]. Furthermore, QDs demonstrate prolonged photostability, rendering them dependable agents for both long-term treatments and imaging applications [105]. Some inorganic quantum dots such as boron quantum dots [106], cadmium telluride quantum dots [107], Ag2S-glutathione quantum dots [108], and black phosphorus quantum dots [109] have shown considerable potential in PDT/PTT due to their excellent optical properties and tunable bandgap. However, their toxicity issues, low environmental stability, poor dispersity, and complex synthesis procedure limit their clinical application. Compared with these quantum dots, carbon-based quantum dots show their advantages such as non-toxicity and high compatibility, good solubility, and are easy to synthesize for more applications. 

## 4. Carbon Dots

As a subclass of carbon-based nanomaterials, carbon dots (CDs) typically exhibit a quasi-spherical structure and measure less than 10 nm in size. Over the years, their remarkable photophysical characteristics, biocompatibility, and minimal toxicity have propelled CDs into the spotlight across various domains, such as bio-imaging [110,111,112], sensors [113,114], and drug delivery [115,116], etc. 

### 4.1. Photosensitizing Properties of Carbon Dots

One particularly intriguing aspect of CDs is their photosensitizing properties, which have garnered significant attention in both fundamental research and practical applications. CDs exhibit inherent photosensitizing properties attributed to their unique chemical composition and surface functionalities [117,118]. The tunable bandgap and surface states of carbon dots enable efficient absorption of light across a broad spectrum, including UV, visible, and NIR regions, thereby facilitating photoexcitation. Upon photoexcitation, carbon dots can undergo various photochemical processes, including electron transfer, energy transfer, and intersystem crossing, leading to the generation of ROS through reactions with molecular oxygen or water. 

Understanding and harnessing the photosensitizing properties of CDs hold immense potential for numerous applications, including advanced antimicrobial therapy through the following mechanisms and/or approaches:

(1) Enhancement of ROS generation: The inherent photoluminescent characteristics of CDs render them promising candidates as photosensitizers. CDs, when appropriately excited, demonstrate the capacity to facilitate robust energy transfer to molecular oxygen, thereby augmenting ROS production. This energy transference is particularly efficient within CDs, owing to their elevated surface area and quantum yield [23].

(2) Chemical functionalization: The adaptable surface of CDs provides a promising pathway for functionalization. Through the introduction of functional groups or additional molecules, the quantum yield of CDs can be significantly improved, rendering them potent photosensitizers. Notably, the incorporation of nitrogen or sulfur into CDs has been demonstrated to enhance their capacity for generating ROS [119,120,121]. Moreover, the conjugation of CDs with conventional photosensitizers can result in synergistic effects, wherein both components synergistically contribute to heightened ROS production, consequently amplifying the efficacy of photodynamic therapy (PDT) [94].

(3) Enhancing specificity to microbial cells: Ensuring the targeted elimination of pathogens while mitigating harm to host cells is a fundamental objective in antimicrobial therapy. CDs present a promising avenue owing to their customizable surface properties. By tethering ligands, peptides, or antibodies onto CDs, they can be tailored to identify and selectively bind to particular microbial structures or receptors. This targeted approach facilitates precise delivery and action. For example, CDs conjugated with antimicrobial peptides exhibit enhanced specificity towards bacterial cells, culminating in focused PDT action [122].

### 4.2. General Synthetic Methods of Carbon Dots

CDs can be generally synthesized using either top-down or bottom-up approaches [123,124]. Top-down methods involve the breakdown of larger carbon structures to produce CDs, typically achieved through techniques such as arc-discharge, laser ablation, and electrochemical oxidation [125]. For instance, laser ablation entails irradiating a carbon target with a high-intensity laser to generate CDs within the resulting soot. In contrast, bottom-up methods utilize smaller carbon precursors to construct CDs. Common techniques include hydrothermal/solvothermal methods, microwave-assisted synthesis, and pyrolysis [126]. In hydrothermal synthesis, for example, small organic molecules undergo a reaction in water at elevated temperatures and pressures, resulting in the formation of CDs.

Each method offers its unique set of advantages. For instance, while the hydrothermal approach might provide better control over the size distribution and is scalable, laser ablation can be faster but might yield CDs with a broader size range. Table 1 compares all general synthetic methods of CDs, outlining their respective advantages and disadvantages [127,128,129,130,131,132,133,134].

**Table 1:** Various “top-down” and “bottom-up” approaches in synthesizing carbon dots (CDs).

Approaches	Synthetic Method	Advantages	Disadvantages	Ref.
Top-down	Laser ablation	Simple and easy to use The size of the CDs can be controlled via adjusting the input laser pulses	Low QY (4.5–18%)	[127]
	Arc discharge	Obtained as by-product from the purification of carbon nanotubes	Low QY (2.3–8.7%) Cumbersome to purify	[128]
	Chemical oxidation	Easy to control the dimension of the CDs by regulating oxidation time and temperature Simple to setup and easy to operate	Time consuming (up to 12 h) Needs further modification	[129]
	Electrochemical oxidation	Economical and environmentally friendly	Time consuming	[130]
Bottom-up	Hydrothermal	Carbon source could be obtained from natural resources or biomass Low-cost apparatus setup Easy to control parameters Large-scale synthesis can be achieved	Product contains impurities such as larger and undissolved particles	[131]
	Combustion	Easy to operate Carbon source could be easily obtained	Low QY (about 1.6%) Broad particle size distribution	[132]
	Microwave irradiation	Rapid and controllable heating	Limitation of large-scale reactions because of small volumes of microwave reactors	[133]
	Pyrolysis	Easy and quick to operate and react	Broad particle size distribution High temperature (180–450 °C)	[134]

## 5. Carbon Dots Used for PDT/PTT Antimicrobial Therapy

### 5.1. Carbon Dots Photosensitizer

#### 5.1.1. Singular Nanosystem

**Figure 2:**
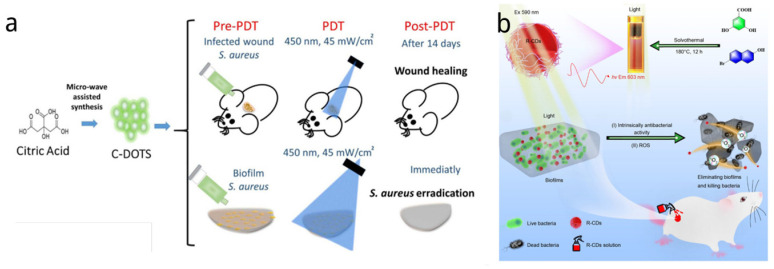
(**a**) Schematic representation of the synthesis of citric acid-derived carbon dots (C-DOTS) and in vivo and in vitro antibacterial photodynamic therapeutic studies. Photoexcited C-DOTS reduced the number of bacterial colonies (log CFU/mL) based on the light dose 450 nm, 40 mW/cm^2^ delivered [135]. (**b**) Synthesis of red-carbon dots (R-CDs) by solvothermal method and its dual effect of intrinsically antibacterial and photodynamic antibacterial effect against MRAB and biofilms [140].

CDs have shown promise as having a good PDT and PTT antimicrobial effect as photosensitizers themselves. For example, Romero et al. synthesized CDs solely from citric acid utilizing a one-pot microwave technique [135]. They assessed the antimicrobial effects of these nanomaterials in combination with light (450 nm at 20 and 40 mW/cm^2^) for both in vitro and in vivo applications (Figure 2a). The in vitro studies examined the bacteria-killing efficacy of illuminated CDs against both suspended and biofilm states of Staphylococcus aureus. Using CDs concentrations of 6.9 and 13.8 mg/mL together with light doses of 20 and 40 J/cm^2^ led to a significant reduction of the bacterial populations. Extrapolating from these findings, in vivo experiments were conducted on mice with *Staphylococcus aureus* (*S. aureus*)-infected wounds. A photodynamic inactivation method, mediated by CDs, was able to diminish bacteria levels by 10^4^ log on the skin lesions. Overall, these studies have demonstrated that CD-facilitated antibacterial photodynamic therapy is an effective and promising treatment modality for infections caused by Gram-positive bacteria, such as wounds infected with *S. aureus*.

Compared to blue emission carbon dots with short-wavelength excitation, carbon dots with red-emission, particularly those emitting in the NIR region (650–900 nm), could offer significant advantages for PDT/PTT such as deeper tissue penetration, reduced photodamage to healthy tissues and enhanced PDT/PTT conversion efficiency. Therefore, designing near-infrared-emitting CDs under long-wavelength excitation should be delved into. The efficient conjugated aromatic π systems and hydrogen generated from aromatic structures was confirmed to cause red shift in the absorption of CDs [118,136,137,138,139]. Liu et al. [140] chose to use 2,4-dihydroxybenzoic acid and 6 bromo-2-naphthol to synthesize red-carbon dots (R-CDs) by a solvothermal method (Figure 2b). R-CDs showed intrinsically antibacterial activities with MIC 32 and 64 µg/mL against Gram-negative bacterium (multi-resistant Acinetobacter baumannii, MRAB) and Gram-positive bacterium (methicillin-resistant Staphylococcus aureus, MRSA), respectively, without light. After 15 min irradiation (590 nm, 30 mW/cm^2^), there were no bacterial colonies found from solid Luria broth agar plates and the bacterial biofilm was destroyed as the R-CD concentration increased, suggesting that R-CDs under irradiation enhanced sterilization efficiency. The wound healing and antibacterial effect of R-CDs in vivo were also evaluated, and the rate of wound healing of treatment groups was much faster than that of control groups.

In another study, N. Sattarahmady and colleagues employed a hydrothermal method to synthesize CDs from ascorbic acid and copper acetate hydrate [141]. These CDs demonstrated a 30% inhibition ratio against both *S. aureus* and MRSA at a concentration of 3.4 μg/mL, even without the application of laser irradiation. Upon exposure to an 808 nm diode laser (0.36 KJ·cm^−2^), inhibition rates rose to 70% at the same concentration of CDs. With intensified laser irradiation, a 100% inhibition ratio was achieved by using a 7 μg/mL concentration of CDs. Additionally, the study explored the mechanisms behind the bactericidal action of CDs, attributing their efficacy to the nanoparticles’ abilities to capture cells and absorb light. Notably, NIR irradiation caused a temperature increase in the solution, resulting in ROS generation and subsequent damage to bacterial cell walls.

CDs could also be modified to realize multiple antimicrobial effects. Chu and co-workers designed and prepared a novel nanocomposite named Cu-RCDs-C35 with triple synergistic sterilization triggered by NIR-emission [142]. The copper functionalized NIR-emitting carbon dots (RCDs) with an enhanced PDT and PTT antibacterial effect were synthesized from citric acid, urea, and cupric chloride dihydrate (CuCl_2_·2H_2_O) using the solvothermal method. Then, the copper functionalized RCDs (Cu-RCDs) were conjugated with a quaternary ammonium compound cocoamidopropyl betaine to promote damage of bacterial membranes. Compared with dark, both RCDs-C35 and Cu-RCDs-C35 could inhibit bacterial growth with laser irradiation (808 nm, 2.0 W/cm^2^, 10 min) as the concentration increased. But, Cu-RCDs-C35 at the same concentration demonstrated more effective antibacterial activity with laser irradiation (808 nm, 2.0 W/cm^2^, 10 min). Meanwhile, the intracellular generation of ^1^O_2_ by Cu-RCDs-C35 was also detected more than that of RCDs-C35, indicating that Cu played an important role in the composite to improve the PDT/PTT effect.

**Figure 3:**
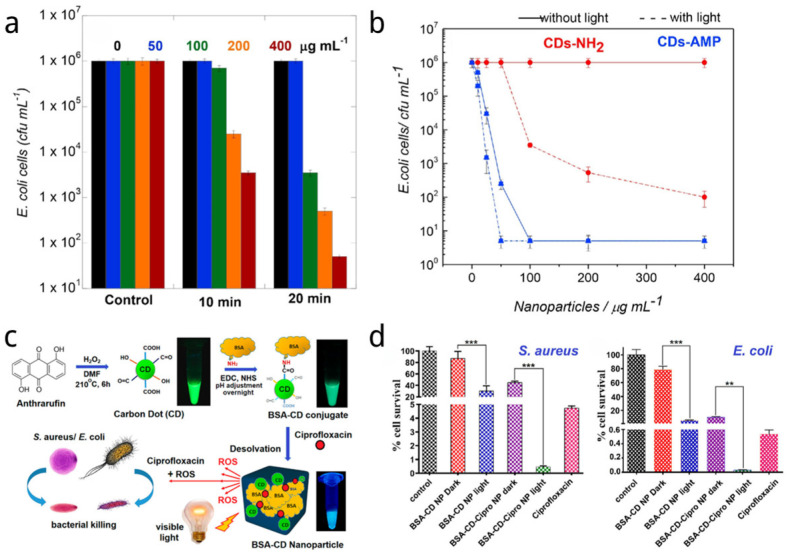
(**a**) Photodynamic efficiency of amine-functionalized CDs (CDs-NH_2_) for the inactivation of *E. coli* upon irradiation at 0.3 W for 10 min and 20 min [143]. (**b**) Influence of the CDs-NH_2_ and CDs-AMP concentration on the treatment efficiency of *E. coli* without (solid lines) and with (dash lines) visible light illumination (20 min, 0.3 W). The error bars represent the standard deviation of three independent experiments [143]. (**c**) Scheme for synthesis of BSA-coated CDs (BSA-CD) for visible-light-induced ROS generation and simultaneous release of ciprofloxacin for antibacterial activity [144]. (**d**) Percentage cell survival of *S. aureus* in the presence of BSA-CD loaded with ciprofloxacin. Percentage cell survival of *E. coli* in the presence of BSA-CD loaded with ciprofloxacin [144]. Data were considered statistically significant and highly significant when *p* < 0.05 and *p* < 0.001, respectively (**, 0.05 < *p* < 0.01; ***, 0.01 < *p* < 0.001).

CDs have also shown their potential to combine with antibiotics to achieve more effective synergistic antimicrobial activity. Amine-functionalized CDs (CDs-NH_2_) were prepared by Boukherrub et al. using citric acid and ethylenediamine via a hydrothermal method [143]. To create CDs-AMP nanostructures, the primary amine groups on the surface of the CDs-NH_2_ were covalently linked to ampicillin (AMP), a common β-lactam antibiotic. The lowest effective dose of the CDs-AMP conjugate (14 μg/mL) inhibited *E. coli* cells more than free AMP alone (25 μg/mL), confirming the enhanced activity of the CDs-AMP conjugate. As shown in Figure 3a,b, the results also demonstrated that exposing CDs-AMP to visible light irradiation further increased its bactericidal effect. Compared to free AMP, the results of this study showed that immobilizing AMP onto CDs improved its stability and antibacterial potency when exposed to visible light.

Mandal et al. used a solvothermal approach to synthesize 1,5-dihydroxyanthraquinone-based CDs that emit green fluorescence, which is shown in Figure 3c [144]. Bovine serum albumin (BSA) was coated onto the surface of the CDs via an amination reaction to boost ROS activity. As depicted in Figure 3c, ciprofloxacin non-covalently interacted with BSA-CDs conjugates to generate drug nanocomplexes. Concurrently, photodynamic antimicrobial chemotherapy worked synergistically with antibiotic drug release to kill 95% of *E. coli* and *S. aureus* at the low concentration of 1.47 μg/mL in their complexes (shown in Figure 3d).

**Figure 4:**
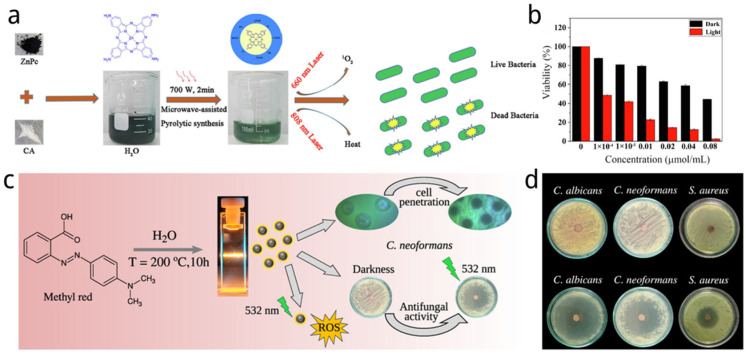
(**a**) Synthetic routes of ZnPc-CQDs for PDT/PTT antibacterial effects [146]. (**b**) Bacterial viability after treatment with different concentrations of ZnPc-CQDs with and without irradiation [146]. (**c**) CDs-MR synthesis scheme with its cell penetration and antimicrobial photoactivation [147]. (**d**) Disk-diffusion (Kirby–Bauer) tests of the antimicrobial activity of CD-MR (120 μg) on MHA plates against *C. albicans*, *C. neoformans*, and *S. aureus* [147].

In addition to the typical small molecular compounds, carbon dots can also be synthesized from molecular photosensitizers. The carbon dots produced in this manner not only exhibit effective photodynamic properties but also overcome the limitations associated with the poor water solubility and stability of molecular photosensitizers [145]. Jiang et al. [146] synthesized a phthalocyanine-CDs (ZnPc-CQD) using zinc (II) tetra-amino-phthalocyanine (ZnPc) and citric acid via microwave pyrolysis (Figure 4a). Compared with ZnPc and CQD synthesized from citric acid, ZnPc-CQD showed better photothermal effects. With irradiation of an 808 nm laser (12 min, 808 nm, 0.5 W/cm^2^), the temperature of ZnPc-CQD could rise to 34.1 °C when the temperature of ZnPc and CQD was just 5.5 °C and 10.0 °C. And, the temperature changes showed concentration dependence, where the temperature of the ZnPc-CQDs solution increased significantly with the increase in concentration. The ZnPc-CQDs showed photothermal conversion efficiencies (η = 27.5%) and ^1^O_2_ generation (ΦΔ = 3.0%). As for photodynamic effects, the generation of ^1^O_2_ from ZnPc-CQDs was a little less than that of ZnPc after 90s irradiation, probably owing to some ZnPc being wrapped by CQD which was consistent with the results of the FT-IR spectrum. This special structure could help to avoid consumption and improve efficiency of ^1^O_2_ since only part of ZnPc would be triggered with light and released, caused by a photothermal effect after the accumulation of ZnPc-CQDs in bacteria. Apart from that, ZnPc-CQDs showed good photoinactivation ability toward both Gram-positive and Gram-negative bacteria upon laser conditions. Compared to single CQD and ZnPc, the ZnPc-CQDs performed obvious bacterial inhibition under laser irradiation and the antibacterial effect was enhanced as the concentration increased (Figure 4b). Specifically, when treated with 0.08 μM ZnPc-CQDs, the survival rate of the *E. coli* and *S. aureus* were 2.40 and 0.50%, respectively. After 660 nm laser irradiation of ZnPc-CQDs, high reactive oxygen species (ROS) could be produced, which also indicates the ZnPc-CQDs could be an ideal material for phototherapy in vivo.

In another study, photosensitizer methyl red azo dye was used to synthesize CD-MR by Ferreira and co-workers via a one-pot hydrothermal method (Figure 4c) [147]. CD-MR showed significant fractions of graphitic nitrogen, contributing to a wide emission range and high singlet oxygen quantum efficiency. The antimicrobial activity of CD-MR was tested against pathogenic microorganisms including Staphylococcus aureus, Candida albicans, and Cryptococcus neoformans using Kirby–Bauer susceptibility tests (Figure 4d). The tests demonstrated antimicrobial activity upon photoexcitation at 532 nm. The growth inhibition of Cryptococcus neoformans due to CD-MR photosensitization was specifically investigated, demonstrating effective ROS generation and strong antimicrobial activity against healthcare-relevant pathogens. The unique properties of these N-doped carbon dots offer a promising approach for targeting drug-resistant pathogenic microorganisms. Moreover, low cytotoxicity against mammalian cells was observed, making them suitable for bioimaging applications.

Table 2 provides a comprehensive summary of the size of CDs, carbon sources, synthesis methods, light sources and irradiation times, applications, and antibacterial doses of carbon dots used as photosensitizers for photodynamic therapy (PDT) and photothermal therapy (PTT) in antimicrobial applications [135,140,141,142,144,146,147,148,149,150,151,152,153,154,155]. 

#### 5.1.2. Nanocomposite

Not only do CDs exhibit PDT/PTT antimicrobial properties on their own, but they also serve as an important component in nanocomposites and/or nanomaterial formulations for PDT/PTT applications (shown in Table 3) [156,157,158,159]. By functioning as both standalone and combinatorial photosensitizing agents, CDs demonstrate versatility in combating microbes through light-activated PDT and PTT approaches.

Carbonized polymer dots (CPD) RF-CPD and RF-CPD/polyurethane (RF-CPDs/PU) composites were designed and fabricated by Markovi’c and co-workers using riboflavin and ethylenediamine as carbon sources [156]. According to the singlet oxygen generation tests and superoxide anions (O_2_^−^) tests, the RF-CPD and RF-CPDs/PU composites both showed high intensity. It is also worth mentioning that these composites were resistive to photo-bleaching since the singlet oxygen production could not be affected by long-term irradiation. They also evaluated the antibacterial effect of RF-CPD and RF-CPDs/PU, and the results showed they both have good bacterial inhibition activity. Apart from that, polyurethane has a high oxygen permeability rate where ROS would diffuse outside the polymer pores and could inhibit bacterial growth effectively.

**Figure 5:**
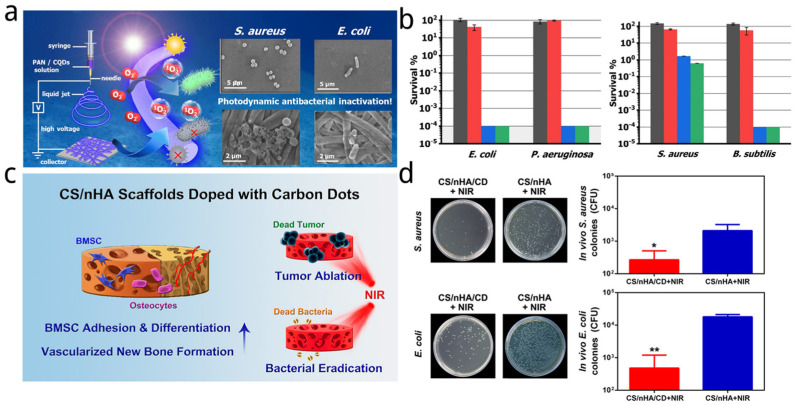
(**a**) Fabrication of PAN-CQDs NFs and the photodynamic inactivation of bacteria upon visible light illumination [157]. (**b**) Photodynamic inactivation studies employing PAN-CQDs NFs against Gram-negative bacteria *E. coli* and *P. aeruginosa* as well as Gram-positive bacteria *S. aureus* and *B. subtilis*. Displayed is the survival rate for the PAN-CQDs-2.5% NFs dark control (dark grey bar) and illuminated PAN NFs light control (red bar) conditions, blue and green bars represent the PAN-CQDs NFs (0.6 and 2.5%) against bacteria, respectively. Studies were performed with a 60 min dark pre-incubation followed by 90 min illumination [157]. (**c**) Design of CS/nHA/CDs scaffolds for enhancing BMSC adhesion and differentiation, promoting vascularized new bone formation, tumor ablation, and bacterial eradication by PTT [158]. (**d**) Number of clinically relevant *S. aureus* (top) and *E. coli* (bottom) bacterial colonies after bacteria from the harvested samples were cultured for 24 h after 1-week treatments in vivo. Each value is the mean ± standard deviation; * *p* < 0.05, ** *p* < 0.01 [158].

Nie et al. [157] designed PAN-CQDs NFs with antibacterial photodynamic inactivation by electrospinning technology (shown in Figure 5a). The carbon dots of composites were synthesized from citric acid and 1,5-diaminonaphthalene via a solvothermal method. Polyacrylonitrile, as a photostable polymer, was used to fabricate PAN-CQDs NF. The Gram-negative bacteria *E. coli* and *P. aeruginosa* were found to be easily susceptible to photodynamic inactivation by the CDs in PAN-CQDs NFs. With 60 min dark pre-incubation followed by 90 min illumination, PAN-CQDs-2.5% and PAN-CQDs-0.6% NFs inactivate *E. coli* and *P. aeruginosa* to the detection limit of 6 log units. And, no difference of bacterial viability was found between the PAN NFs with illumination (PAN NFs light control) and PAN-CQDs-2.5% NFs without illumination (dark control), which both further determined a photodynamic inactivation effect towards Gram-negative bacteria and illustrated that PAN-CQDs-2.5% NFs has no dark toxicity. For Gram-positive bacteria, PAN-CQDs-0.6% and PAN-CQDs-2.5% NFs inactivate *S. aureus* by 98.3 and 99.4%, while the PAN-CQDs-0.6% and PAN-CQDs-2.5% NFs could inhibit 99.9% B. subtilis (Figure 5b). Followed the detection of ROS (^1^O_2_), the mechanism of antibacterial photodynamic inactivation by the CQDs prepared here is consistent with the photodynamic generation of biocidal singlet ^1^O_2_ and suggested that the PAN-CQDs NFs could be Type II photodynamic materials.

In another study, a novel CD-doped chitosan/nanohydroxyapatite (CS/nHA/CD) scaffold was developed by Lu et al. (Figure 5c) [158]. The carbon dots embedded were synthesized from citric acid and glycine by a domestic microwave oven (800 W). By integrating with CDs, the scaffold performed an enhanced effect of bone marrow-derived stem/stromal cells (BMSC) adhesion and differentiation and vascularized new bone formation. Moreover, the CS/nHA/CD scaffolds showed good antibacterial effect which could be enhanced after NIR irradiation (808 nm, 1 W/cm^2^). Specifically, the CS/nHA/CD + NIR group (contained 1 mg/mL CDs) had significantly higher antibacterial activity towards pathogenic *S. aureus* and *E. coli* (with an antibacterial rate of 99 and 97%, respectively, whereas the antibacterial rates were approximately 75% in the other groups (Figure 5d).

### 5.2. CDs as Booster in Combination with Other Photosensitizing Agents

By functioning as both standalone and combinatorial photosensitizing agents, CDs demonstrate versatility in combating microbes through light-activated PDT and PTT approaches. 

Dong et al. [160] combined CDs with photosensitizers methylene blue (MB) or toluidine blue (TB), leading to enhanced synergistic PDT antibacterial effects. The combination of 5 µg/mL CDs with 1 µg/mL MB or TB resulted in a complete inhibition of bacterial growth, indicating a synergistic interaction between the components. This was a significant improvement over the effects observed with MB or TB alone. The synergistic effects were quantitatively confirmed through broth microdilution checkerboard methods and isobologram analyses, with the fractional inhibitory concentration index values supporting synergy. Intracellular ROS measurements showed that the combination treatments significantly increased ROS production compared to treatments with MB or TB alone. This increased ROS generation is a key factor in the enhanced antimicrobial efficacy observed in the study. The reason for that could be summarized as follows: (1) MB and TB could increase cellular penetration, (2) CDs could be helpful to improve solubility of their integral combination and thus improve uptake/localization and target delivery, (3) The combination of CDs and photosensitizers could increase overall intracellular ROS via fluorescence resonance energy transfer (FRET) mechanisms. 

**Figure 6:**
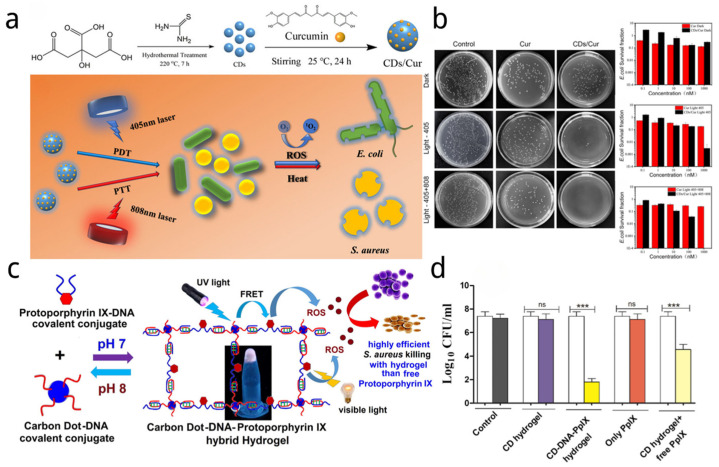
(**a**) Synthesis of the CDs/Cur nanocomposite and bactericidal activities of CDs/Cur upon dual wavelength (405 + 808 nm) illumination [36]. (**b**) Agar plate photographs of *E. coli* treated with Cur or CDs/Cur, respectively, were taken under (non) light irradiation conditions. The corresponding dependence of *E. coli* survival fraction on the concentration of Cur and CDs/Cur was measured, respectively, under non-light, 405 nm light, and 405 + 808 nm light. Values are means ± standard deviation (SD) (n = 3) [36]. (**c**) Design of hybrid hydrogel derived from carbon dots (CDs), protoporphyrin IX (PpIX), and DNA [37]. (**d**) Plate count assay for *S. aureus.* cell survival with CD-DNA-PpIX hydrogel (PpIX:1 mM) exposed to UV light [37]. Each value is the mean ± standard deviation; *** *p* < 0.001, ns: non-significant.

A nano-PS system (CDs/Cur) containing carbon dos and natural photosensitizer curcumin (Cur) was constructed by Yan et al. [36] Carbon dots were synthesized from citric acid and thiourea by hydrothermal treatment and then bound with curcumin by electrostatic attraction (Figure 6a). Upon combined near-infrared (808 nm) and 405 nm visible dual-wavelength irradiation, CDs/Cur could simultaneously generate ROS and cause a moderate temperature increase, triggering synergistic antibacterial effects against both Gram-positive and Gram-negative bacteria. As shown in Figure 6b, when treated with 405 nm laser irradiation, 99.9% of *E. coli* would be killed by 1 μM CDs/Cur, while the death rate of *E. coli* increased to 100% for 1 μM CD/Cur when combined with near-infrared light. However, the inhibition effect of Cur against *E. coli* was almost the same (death rate < 90%) under double wavelength irradiation. Similarly, for *S. aureus*, just 0.1 nM CDs/Cur could kill 100% of them under the combination of near-infrared and 405 nm visible dual-wavelength irradiation. However, the curcumin still exhibited negligible antibacterial effect against *S. aureus*.

Kumari et al. [37] designed a novel hydrogel system using CDs-DNA as a crosslinking agent and FRET donor to promote PpIX activation, which in turn generated reactive oxygen species (ROS) for killing Gram-positive bacteria and Gram-negative bacteria (shown in Figure 6c). The FRET production of CDs and the PDT effect of PpIX under visible light can generate ROS together, which could considerably improve photodynamic activity. For instance, as shown in Figure 6d, after UV irradiation for 2.5 min, the treatment with 10 mM CDs-DNA-PpIX hydrogel resulted in a reduction of more than 4.5 log (>99.99%) in *S. aureus* cell count, whereas the mixture of CDs hydrogel with free PpIX showed a reduction of more than 2.8 log (>99.8%). This effect was dose-dependent, as under similar conditions, 2.5 mM of CDs-DNA-PpIX hydrogel and CDs hydrogel mixed with free PpIX, respectively, exhibited more than 1.4 log (>95%) and more than 1 log (>90%) reduction in cell viability. Therefore, the CDs-DNA-PpIX hybrid hydrogel and the mixture of CDs hydrogel with free PpIX demonstrated notable antimicrobial activity upon UV light exposure. However, the CDs hydrogel alone and PpIX did not show significant killing effects on *S. aureus* cells after UV exposure. Additionally, the antimicrobial effect of the hydrogel mixtures was tested under visible light irradiation. After 5 min of visible light exposure, the CDs-DNA-PpIX hydrogel achieved a reduction of more than 4.8 log (>99.99%) in the *S. aureus* cell count, whereas the CDs hydrogel mixed with free PpIX showed a reduction of more than 2.5 log (>99.5%). Compared to cells not treated with hydrogel, a free PpIX solution (at the same concentration used in the hydrogels) under visible light displayed more than a 1.2 log (>93%) reduction in cell viability. The experiments clearly demonstrated a more efficient cell killing efficiency when hydrogels were involved. 

Apart from that, the hydrogel platform could demonstrate long-term release of this composite and ROS could be generated slowly and consistently, thus the Gram-positive bacteria (*S. aureus*) could be inhibited or even killed continuously. Specifically, precisely controlling the sol-gel transition of the hydrogel by adjusting the pH value enables the continuous release of PpIX and the generation of ROS for a duration of more than 10 days.

There are other studies about carbon dots as boosters in photosensitizer nanocomposites for PDT/PTT antimicrobial effects [35,36,37,161], as listed in Table 4.

## 6. Conclusions and Future Perspectives

In recent years, carbon dots (CDs) have garnered significant attention in various biomedical applications such as biosensing, bioimaging, antimicrobial activities, phototherapy, and drug delivery. CDs exhibit potential as alternative therapeutic agents or as adjuvants to antibiotics, thereby mitigating the issue of drug resistance. This paper explores the role of CDs as photosensitizers, both independently and in conjunction with other photosensitizers, in photodynamic therapy (PDT) and photothermal therapy (PTT). Initially, the principles of PDT and PTT were introduced, followed by a summary of different categories of photosensitizers, highlighting new sources with high photoexcitation activity for CDs. Subsequently, the photoactivated properties of CDs were examined to elucidate their role in antimicrobial therapy. CDs enhance ROS generation due to their high surface area and quantum yield, making them effective photosensitizers. Chemical functionalization and conjugation with other photosensitizers further improve their ROS production, and PDT efficacy and customizable surfaces of CDs enable targeted antimicrobial therapy by binding to specific microbial structures. Additionally, the synthesis methods of CDs were also reviewed with a comparison of the advantages and disadvantages of different methods, providing valuable references for carbon dot synthesis research. Furthermore, CDs as a photosensitizer and booster in nanosystems for PDT/PTT antimicrobial therapy were reviewed, indicating the potential of CDs for creating more effective antibacterial nanomaterials and emphasizing the combination of CDs with other photosensitizer molecules and the integration of CDs into various nanosystems. The information of carbon source, synthesis method, light source, and bacteria species inhibited by carbon dots were summarized, providing reference for related research. Carbon dots (CDs) as a photosensitizer or with a role to boost other photosensitizers have emerged as a promising tool in antimicrobial photodynamic and photothermal therapy, yet they face numerous challenges. One significant issue is the inconsistent absorption wavelengths and variable quantum yields of CDs, leading to unpredictable ROS production [162]. Size and shape variability, surface functional groups, synthesis conditions, and doping elements are factors contributing to inconsistent absorption wavelength and quantum yields. To address this problem, standardizing synthesis conditions and post-synthesis modification can help achieve production of CDs with uniform sizes and shapes, leading to more consistent absorption properties [152,163,164]. This will ensure that the CDs reliably produce reactive oxygen species (ROS) needed for effective antimicrobial action. Incorporating stable elements such as nitrogen or phosphorus into the CD structure can help achieve more reliable ROS generation [36,165,166]. Functionalization of CDs, while necessary to enhance biocompatibility or targeting ability, can alter their beneficial properties, potentially reducing their antimicrobial efficacy. This issue can be mitigated by using protective coatings such as polyethylene glycol (PEG) or silica that preserve the functional properties of CDs while improving their stability and biocompatibility [167,168,169]. Such coatings can shield the CDs from environmental factors that might otherwise degrade their performance.

Another critical concern is the toxicity associated with the photodegradation of CDs [170,171]. While short-term light exposure (typically 10–30 min) is usually sufficient for antimicrobial action and is unlikely to cause significant photodegradation, the exact effects of this brief exposure on normal cells remain underexplored. Current research on the photodegradation toxicity of CDs is limited, and it remains unclear whether the degradation products generated during such short exposure times can induce toxicity in normal cells. This uncertainty highlights the need for further studies to evaluate the safety of CDs under typical antimicrobial treatment conditions. To reduce the risk of photodegradation toxicity, surface functionalization with photostable coatings such as silica or PEG can protect CDs from photodegradation while allowing efficient ROS generation. Additionally, using biodegradable coatings can minimize the long-term toxicity associated with CD degradation within the body. For external application, the skin acts as a natural barrier, limiting the penetration of CDs into deeper tissues and reducing the risk of systemic toxicity. For internal applications, targeted delivery systems are crucial. CDs need to be delivered specifically to infection sites to minimize exposure to normal cells. Utilizing nanocarriers such as liposomes or polymeric nanoparticles or incorporating targeting ligands that bind specifically to microbial cells, could probably enhance the selectivity and efficacy of the treatment [23,172]. Controlled light delivery methods, including endoscopic techniques or implantable light sources, can further improve treatment specificity.

In summary, carbon dots hold considerable promise for use in photodynamic and photothermal antibacterial therapies due to their unique optical properties, biocompatibility, and functionalizability. However, to fully realize their potential in clinical applications, continued research and development are essential. Addressing the bottlenecks and challenges, such as optimizing synthesis methods, improving stability and biocompatibility, and ensuring effective light penetration, will be crucial for advancing CDs in antimicrobial therapeutics. Further studies on the photodegradation toxicity of CDs are particularly needed to clarify their safety profile and guide the development of safer, more effective antimicrobial treatments. 

## Figures and Tables

**Table 2:** Carbon dots, as photosensitizers, used for PDT/PTT antimicrobial therapy.

Name	Size of CDs (nm)	Sources of CDs	Synthetic Method of CDs	Light Source and Power and Time	Application	Strain and Dosage	Ref.
C-DOTS	1.5–4.5	Citric acid	Microwave	450 nm; 40 mW/cm^2^	PDT	*S. aureus* 13.8 mg/mL	[135]
R-CDs	3.31	2,4-dihydroxybenzoic acid and 6bromo-2-naphthol	Solvothermal	590 nm, 30 mW/cm^2^	PDT	MRAB and MRSA 30 µg/mL^−1^	[140]
C-dots	12 ± 1	Ascorbic acid and copper acetate hydrate	Hydrothermal	808 nm diode laser 0.36–0.84 KJ/cm^2^	PTT	*S. aureus* and MRSA 7.0 μg/mL^−1^	[141]
Cu-RCDs-C35	8.8	Citric acid, urea, and cupric chloride dihydrate (CuCl_2_ 2H_2_O)	Solvothermal	808 nm irradiation (2.0 W/cm^2^, 10 min)	PDT and PTT	*E. coli* and *S. aureu* 800 μg/mL	[142]
BSA-CDs	5	1,5-dihydroxyanthraquinone	Hydrothermal	Visible light (100 W, 1 h, 30 cm distance)	PDT	*S. aureus* and *E. coli*	[144]
ZnPc-CQDs	5.2 ± 1.2	ZnPc, citric acid	Microwave-assisted	660 + 808 nm, 12 min, 0.5 W/cm^2^	PDT and PTT	*E. coli* and S. aureu, 0.08 μM	[146]
CD-MR	3.3	Methyl red azo dye	Hydrothermal method	LED 532 nm, 10 mW	PDT	*C. albicans*, *C. neoformans*, and *S. aureus*	[147]
BAPTCDs	3–5	O-phenylenediamine and D-Glu	Hydrothermal	808 nm, 1.5 W/cm^2^ for 10 min	PTT	*S. aureus* and *E. coli* 200 μg/ml	[148]
DHLA@MCDs	2–6	Edible mushroom	Oven	Visible LED light 2.70 mW/cm^2^	PDT	*E. coli*	[149]
CDs-AMP	2	Citric acid and ethylenediamine	Hydrothermal	LED light (365 nm) 3 V/3 W, 1 h	PDT	*E. coli* and Salmonella 5 µM	[150]
BrCDs	0.7	Natural gas, HBr	Halogenation	Ultraviolet lamp (365 nm) 3 mW	PDT	*Listeria* monocytogenes, *S. aureus* and *E. coli*.	[151]
EDA-CDs/EPA-CDs	4–5	Carbon—nano-powders	Hydrothermal	400–800 nm light bulb 36 W, 12 V	PDT	*Bacillus subtilis*	[152]
FCDs	1–10	Glucosamine hydrochloride and mphenylenediamine	Microwave-assisted	Blue-LED strip lights (460 nm) 24 W, 12 V	PDT	*Klebsiella* *pneumoniae*, *Pseudomonas* *aeruginosa*, *E. coli* and *S. aureus* 200 µg/mL	[153]
Cur-NRCQDs	3.83	Ctric acid, neutral red, curcumin	Hydrothermal method	Xenon light (400–450 nm)	PDT	*S. aureus* and *E. coli*, 10 mM and 15 mM	[154]
ST-JHCQDs	10.7	Turmeric extract	Hydrothermal method	405 nm (20 mW/cm^2^ for 30 min)	PDT	*S. aureus* and *E. coli* 5 mg/mL	[155]

**Table 3:** Carbon dots, as photosensitizers, integrated into other nanocomposites and/or nanomaterial formulations, for PDT/PTT antimicrobial therapy.

Name	Size of CDs (nm)	Sources of CDs	Synthetic Method of CDs	Light Source and Power and Time	Application	Strain and Dosage	Ref.
RF-CPDs/PU	35.2 ± 2.1	Riboflavin, ethylenediamine	Hydrothermal method	Blue light (power 3 W) for 30, 60, and 120 min	PDT	*E. coli* and *S. aureus.*	[156]
PAN-CQD	-	Citric acid and 1,5-diaminonaphthalene	Solvothermal	Xe lamp, λ ≥ 420 nm, 12 cm sample distance, 500 W for 1.5 h	PDT	*Escherichia coli* and *Pseudomonas aeruginosa*	[157]
CS/nHA/CD scaffolds	5	Citric acid and glycine	Domestic microwave oven (800 W)	808 nm, 1 W/cm^2^ for 10 min	PTT	*S. aureus* and *E. coli*, 1 mg/mL	[158]
BPs@CQDs	1–10	Tellurocystine	Solvothermal	808 nm, 1.5 W/cm^2^	PDT and PTT	*S. aureus* and *E. coli*	[159]

**Table 4:** Carbon dots, as booster in photosensitizer nanocomposites, for PDT/PTT antimicrobial therapy.

Name	Sources for CDs	Photosensitizers	Synthetic Method	Light Source and Power and Time	Application	Strain and Dosage	Ref.
CDs-Cur	Citric acid and ethylenediamine	Curcumin	Microwave	800 nm (500 mW/cm^2^) 405 nm (200 mW/cm^2^)	PDT/PTT	*S. aureus*/*E. coli* 1 µM	[35]
CDs/Cur	Citric acid and thiourea	Curcumin	Hydrothermal	800 nm (500 mW/cm^2^) 405 nm (200 mW/cm^2^)	PDT/PTT	1 µM and 0.1 nM CD/Cur for *E. coli* and *S. aureus* respectively	[36]
CD-DNA-PpIX hybrid hydrogel	Citric acid and Branched Polyethylenimine	PpIX	-	UV lamp (302 nm)	PDT	*S. aureus*	[37]
cur-GQDs	Coal and curcumin	Curcumin	Solvothermal method	405 nm LEDs 30 J/cm^2^	PDT	*Pseudomonas aeruginosa*, MRSA, *E. coli*, and *Candida albicans*.	[161]

## Data Availability

Not applicable.
